# A Reliability-Based Multisensor Data Fusion with Application in Target Classification

**DOI:** 10.3390/s20082192

**Published:** 2020-04-13

**Authors:** Gabriel Awogbami, Abdollah Homaifar

**Affiliations:** Autonomous Control & Information Technology (ACIT) Institute, Electrical and Computer Engineering Department, North Carolina A & T State University, Greensboro, NC 27411, USA; giawogba@aggies.ncat.edu

**Keywords:** multisensor, reliability, classification, belief function, evidence theory, data fusion

## Abstract

The theory of belief functions has been extensively utilized in many practical applications involving decision making. One such application is the classification of target based on the pieces of information extracted from the individual attributes describing the target. Each piece of information is usually modeled as the basic probability assignment (BPA), also known as the mass function. The determination of the BPA has remained an open problem. Although fuzzy membership functions such as triangular and Gaussian functions have been widely used to model the likelihood estimation function based on the historical data, it has been observed that less emphasis has been placed on the impact of the spread of the membership function on the decision accuracy of the reasoning process. Conflict in the combination of BPAs may arise due to poor characterization of fuzzy membership functions to induce belief mass. In this work, we propose a multisensor data fusion within the framework of belief theory for target classification where shape/spread of the membership function is adjusted during the training/modeling stage to improve on the classification accuracy while removing the need for the computation of the credibility. To further enhance the performance of the proposed method, the reliability factor is deployed not only to effectively manage the possible conflict among participating bodies of evidence for better decision accuracy but also to reduce the number of sources for improved efficiency. The effectiveness of the proposed method was evaluated using both the real-world and the artificial datasets.

## 1. Introduction

An integral component of an effective and efficient defense system to aid the commander in situational awareness of the battlefield is target classification. The task of classifying targets into a predefined set of classes depend on a group of features or attributes that characterize the different categories. Sensors such as radar, infrared (IR) camera, and electronic support measure (ESM) are often deployed to acquire relevant information regarding the different attributes [1,2]. Attributes may include signature and kinematic features such as speed, acceleration, altitude, radar cross-section (RCS), shape, length, transmission frequency, pulse repetitive frequency interval (PRI) [1,2].

Classification of a target requires data about the different attributes. The information extracted from the data is usually characterized by uncertainty due to ambiguity, imprecision, vagueness, incompleteness, noise, and conflict [3,4,5]. This uncertainty corrupts the quality of the information fusion system. Consequently, how to effectively and efficiently deal with uncertainty has become a topic of interest among researchers in the field of information fusion systems. Multisensor data fusion can effectively address this problem. Dealing with uncertainty through data fusion provokes three fundamental problems of (1) representation of uncertain information (2) aggregation of two or more pieces of uncertain information and (3) making a reasonable decision based on the aggregated pieces of information [6]. Different mathematical theories of uncertainty, such as probability theory, fuzzy set theory, possibility theory, and belief theory, can be used to tackle these problems. The framework of belief theory, also known as the Dempster Shafer (DS) theory, was first introduced by Dempster in [7] and later extended in [8] by Shafer. The theory of belief functions has an established nexus with the probability theory, the possibility theory, and by extension, the fuzzy set theory. The DS theory offers provision for the representation of ignorance. The DS theory of belief functions has been widely accepted as a powerful formalism for modeling and reasoning under uncertainty.

Implementation of the framework of belief theory entails three building blocks: the modeling, the reasoning, and decision making. The modeling involves the representation of historical data to build the belief function model. The reasoning stage is characterized by the generation, analysis, and the combination of belief functions from the various uncertain evidence sources. The transformation of the fused mass into the probability distribution and the application of an appropriate decision rule is the focus of the decision-making block. How to generate the mass function, also known as the basic probability assignment is an open problem [9]. There is a rich collection of articles to address this problem. At the emergence of the theory, in its various deployments, masses are assigned based on expert opinion [2]. In [9], masses are assigned based on the normal distributions. Fuzzy membership functions have been utilized in [10,11,12,13]. The heart of the reasoning stage is the DS rule of combination. The DS rule of combination can be used to fuse multiple pieces of evidence. However, the DS combination rule suffers a major setback of counter-intuitive results when it is required to aggregate pieces of evidence that are highly conflicting with one another. This phenomenon was first pointed out by Zadeh in [14], and it has since become another open issue. An attempt to address this problem has given birth to two schools of thought among researchers [15]. One school of thought believes that the high conflict is due to a problem with the original combination rule. Consequently, they proposed a modification of the traditional DS rule of combination. Some of the significant contributions in this category can be found in [16]. This approach is such that the conflicting masses are transferred to the universal set. According to [17], the resulting masses obtained from the combination of conflicting sets are assigned to the union. Philippe Smets in [18] proposed an alternative method where the conflicting mass is assigned to the empty set. The other school of thought attributed the high conflict to bad evidence or a faulty sensor. As a result, they believe in the modification of the original basic probability assignment (BPA) before the application of the traditional DS rule of combination. These methods are similar to the discounting measure proposed by Shafer in [8]. A simple average combination rule was proposed in [19]. In [20], it was argued that Murphy’s approach does not account for the relationship among the participating bodies of evidence; consequently, a weighted combination approach based on the distance of evidence was proposed. Also, in [21], Zhang proposed a weighted evidence combination that is capable of identifying and reducing the impact of the conflict. The method explores the cosine similarity among the Pignistic transformations of the various belief functions. Leveraging the strength of methods proposed in [19,20] in terms of simplicity and taking into consideration the relationship among participating belief functions, a new combination method based on the average/consensus belief function was proposed in [22].

In [11], a target classification algorithm was proposed that employed fuzzy membership functions to generate belief functions where belief functions are combined using the traditional DS rule of combination. The method did not account for possible conflict among the participating BPA, despite the availability of information regarding the BPA, which can be harnessed to mitigate the effect of conflict. To deal with conflict, in [12], the fusion stage takes into consideration the credibility of each piece of evidence before the combination. The credibility is based on information contained in the BPAs. Also, in [13], a fusion method termed reliability credibility Dempster Shafer rule of combination (RCDSRC) was introduced that utilizes the credibility of each BPA as well as the reliability of the source for proper adjustments of BPAs prior to the DS fusion. It has been observed that in [12,13], the spread of the membership function was set to 2 standard deviation from the mean. According to [23], conflict in the combination of evidence can be attributed to three main factors: (1) abnormal measurement by sensor usually a direct consequence of sensor defect and poor calibration, (2) improper belief function model due to poor estimation of the likelihood function and inappropriate selection of metric for the distance-based method, and (3) Large number of information sources. The membership functions are used to estimate the likelihood of the various classes; this means improper characterization of the fuzzy membership functions may induce conflicts. The idea behind this study is that by adjusting the spread of the membership function, we can improve on the decision accuracy of the method proposed in [13].

In this study, we propose a reliability-based multisensor data fusion, which is coined as *reliability-based Dempster Shafer rule of combination (RDSRC)*, within the framework of the belief theory for target classification where shape/spread of the membership functions is adjusted during the training/modeling stage. Only the reliability is used to assign weights to the various information sources. The proposed method does not utilize credibility. Since every attribute (information source) of the unknown target produces a local declaration in the form of a belief function, calculation of the credibility for each belief function for every query target will incur additional overhead costs of the reasoning process. Besides the computational requirement, the credibility based on distance or similarity measure is with the assumption that the majority of the belief functions are reliable.

The proposed method is closely related to the work in [24]. However, they are different in the following respects: in this approach, we use triangular membership functions to model the historical data regarding the different attributes of the various target classes as opposed to the Gaussian membership function used in [24]. The reliability in this approach was calculated using an evaluation criterion based on the concordance index, while the Jaccard index was utilized in the determination of the static reliability in [24]. The spread of membership function is adjustable in our proposed method while it is fixed in [24]. Although the tuning of the spread of the membership function in the proposed method introduces additional overheads, it is only incurred offline. In [13,24], credibility/dynamic reliability is calculated at the reasoning phase, which creates an extra cost for on-line identification. The method of generating the BPA is different from the one used in [24]. This work is basically an extension/modification of [13]. The major contributions of the newly proposed method are summarized as follows.

1. We introduced a tuning parameter for the likelihood estimation function and demonstrated its impact on the decision accuracy of the classification system.
2. We proposed the average pairwise discordance index (APDI) as a selection criterion to reduce the number of evidence sources before the deployment of the DS framework.
3. Three real-world and one artificially generated datasets were used to show the performance of the proposed method in terms of accuracy.

The rest of the paper is organized as follows: The basic preliminaries are briefly discussed in Section 2. In Section 3, the proposed reliability based multisensor data fusion with application in target classification is presented. The focus of Section 4 is to show the effectiveness of the proposed approach on both the real and artificial datasets. The conclusion is contained in Section 5.

## 2. Preliminaries

### 2.1. Dempster-Shafer Theory (DST)

The Dempster Shafer (DS) theory, often referred to as the theory of belief functions, was originally introduced by Dempster in [7] and later developed by Shafer in [8]. The theory of belief functions allows probabilities to be assigned to subsets instead of only mutually exclusive singletons. It can model uncertainty better than the probability theory [16]. The basics of the DS theory include the frame of discernment, functions, the DS rule of combination, and the probability transformation.

#### 2.1.1. Frame of Discernment

Let Ω, a set of *M* mutually exhaustive and exclusive hypotheses be defined as

(1) $$\Omega =\{\omega_{1},\omega_{2},\ldots ,\omega_{M}\}$$

Ω is known as the frame of discernment. A power set $2^{\Omega }$ is the set of all possible subsets of Ω.

#### 2.1.2. Functions

For all A,B⊆Ω, evidence can be represented by functions which include: mass function, belief functions, and plausibility functions [25].

Mass function $m:2^{\Omega }\to \left[0,1\right]$ satisfies the following conditions:

(2) $$\sum_{A\subseteq \Omega }m\left(A\right)=1$$

m(∅)=0

Belief function $Bel:2^{\Omega }\to \left[0,1\right]$ is defined as:

(3) $$Bel\left(A\right)=\sum_{B\subseteq A}m\left(B\right)$$

Plausibility function $Pl:2^{\Omega }\to \left[0,1\right]$ is defined as

(4) $$Pl\left(A\right)=\sum_{A\cap B\neq \emptyset }m\left(B\right)$$

#### 2.1.3. The DS Rule of Combination

To fuse evidence from multiple independent sources, the DS rule of combination is used. Suppose $m_{1}$ and $m_{2}$ are two mass functions obtained from two independent sources on the same frame of discernment Ω. The combined mass is defined as [8]

(5) $$m\left(A\right)=\frac{\sum_{B\cap C=A}m_{1}\left(B\right)m_{2}\left(C\right)}{1-\sum_{B\cap C=\emptyset }m_{1}\left(B\right)m_{2}\left(C\right)}$$

∀A,B,C⊆Ω and A≠∅.

#### 2.1.4. Probability Transformation

The mass function obtained after the application of the DS combination rule is not adequate for decision making, consequently probability transformation is required to obtain probability values from the fused mass function. The Pignistic probability function introduced in [18] is often applied and it is formally defined as

(6) $$BetP\left(\left\{A\right\}\right)=\sum_{A\subseteq B}\frac{\left|A\cap B\right|}{\left|B\right|}m\left(B\right)$$

### 2.2. Fuzzy Set Theory

Fuzzy set theory is a theoretical framework for handling imperfection in data [26]. Its concept is built on fuzzy sets to model uncertainty due to imprecision and vagueness. A fuzzy set is described by a membership function which allows an object to belong to different classes with varying degree of membership ranging from [01] [26].

#### 2.2.1. Fuzzy Membership Function

The membership function μ is the mapping of each element *x* to a value μ(x) on [0,1]. Although, there are several membership functions, the commonly used ones are Gaussian, triangular and trapezoidal functions. Gaussian membership function for set *A* is defined as

(7) $$\mu_{A}\left(x\right)=e^{-\frac{x-{\bar{x}}_{ij}}{2\sigma^{2}}}$$

A triangular fuzzy number *A* can be described by the triplets (a,b,c) with the membership value defined as

(8) $$\mu_{A}\left(x\right)=\left\{\begin{array}{cc} 0, & x\leq a\\ \frac{x-a}{b-a}, & a<x\leq b\\ \frac{c-x}{c-b}, & b<x\leq c\\ 0, & x>c \end{array}\right.$$

Nonetheless the structure of the triangular fuzzy number is not as smooth as the Gaussian membership function, it is simpler and easier to use.

#### 2.2.2. Type−2 Fuzzy Sets

**Figure 1:**
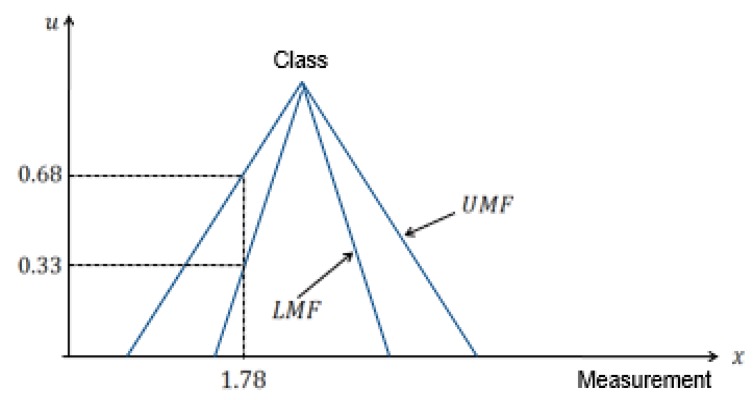
Representation of Type−2 fuzzy sets with primary membership function.

**Figure 2:**
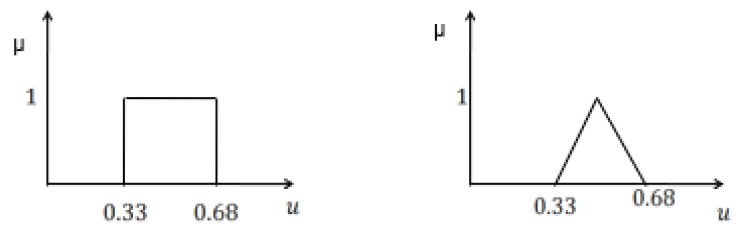
Representation of Type−2 fuzzy sets with secondary membership function.

The selection of the right type of membership function is one of the major challenges of using Type−1 fuzzy membership function for data and uncertainty representation. Type−2 fuzzy set can be used to solve the problem. The fact that different spreads of the membership function will produce different accuracy corroborates one of the reasons for the use of Type−2 fuzzy sets to model uncertainty about the appropriate type of membership function. Type−2 fuzzy set can be viewed as a collection of many embedded Type−1 fuzzy sets [27,28]. The membership value in a Type−2 fuzzy set is itself a fuzzy set. The traditional Type-1 fuzzy set is two dimensional (2D), however, the Type−2 fuzzy set is three dimensional (3D) to include element, primary membership value and secondary membership value denoted by *x*, *u* and, μ respectively as shown in Figure 1 and Figure 2. The area between the lower membership function (LMF) and the upper membership function (UMF) is termed *footprint of uncertainty (FOU)* [28]. We provided a brief introduction to Type−2 fuzzy set because it forms the basis of the intuition for varying the spread factor of the membership function to model uncertainty about the membership function.

It can be observed that Type−2 fuzzy set can represent and deal with uncertainty associated with membership function by leveraging the additional degree of freedom provided by the newly introduced third dimension and the footprint of uncertainty.

#### 2.2.3. Similarity Between Fuzzy Sets

**Figure 3:**
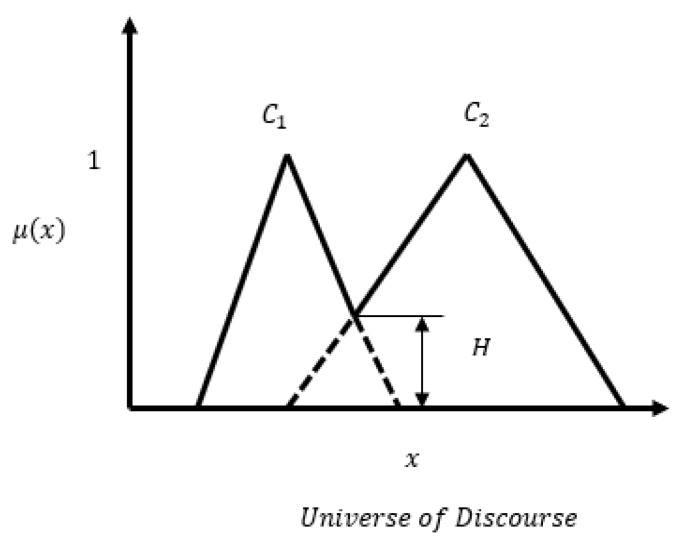
Concordance and Jaccard similarity indices.

In the literature, there are several measures of similarity between two fuzzy sets. Jaccard index and concordance index are of interest to us in this context. As shown in Figure 3, let us assume that $\mu_{1}$ and $\mu_{2}$ are two Gaussian membership functions for class $C_{1}$ and class $C_{2}$. The concordance index $S_{C}$ and Jaccard index $S_{J}$ are defined as [29]

(9) $$S_{C}\left(C_{1},C_{2}\right)=sup\;\min_{x}\left\{\mu_{1}\left(x\right),\mu_{2}\left(x\right)\right\}$$

(10) $$S_{J}\left(C_{1},C_{2}\right)=\frac{\int_{R}min\left(\mu_{1},\mu_{2}\right)dx}{\int_{R}max\left(\mu_{1},\mu_{2}\right)dx}$$

## 3. Proposed Method

Target classification problem can be formulated in the same way as the general data classification problem as follows. Let $X=\{x_{1},x_{2},\ldots ,x_{n}\}$ be a set of *n* training samples with corresponding class labels $\left\{y_{1},y_{2},\ldots ,y_{n}\right\}$, $x_{k}$ is a N−dimensional attribute vector with class label $y_{k}$, where $y_{k}\in \Omega =\left\{C_{1},C_{2},\ldots ,C_{M}\right\}$, which is a set of *M* classes. Suppose in a target classification system, a set of N−sensors $\left\{s_{1},s_{2},\ldots ,s_{N}\right\}$ measuring different attributes of the target produces a collection of basic probability of assignment (BPA) defined as $\left\{m_{1},m_{2},\ldots ,m_{N}\right\}$. The primary goal of a target classification system is to assign the unknown target x to one of the members of the frame of discernment based on the combination of different pieces of evidence induced by the different attribute measurements.

**Figure 4:**
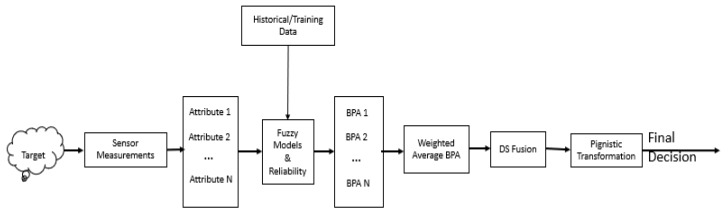
The flowchart of the proposed multisensor data fusion for target classification.

In [23], it was asserted that the conflict within the framework of belief theory could be attributed to improper likelihood estimation function. Since fuzzy membership function is employed as the likelihood estimation model which is parameterized by the spread factor, poor characterization using the spread factor γ may result in high conflict and consequently, a degradation in the performance of the reasoning process. The motivation for this study was triggered by the application of interval Type−2 fuzzy set for the representation of uncertainty. An interval type 2 fuzzy set is a form of Type2 fuzzy set with uniform secondary membership function. Interval type2 fuzzy set is characterized by upper and lower membership functions. It was discovered that using the lower membership function at the modeling stage did not yield the same value of accuracy as utilizing the upper membership function. Moreover, the only difference between the two is the spread/width. This gives the insight that by varying the spread parameter, we can actually improve on the performance of the proposed method in terms of accuracy. In this work, the theory of belief functions is being proposed as a multisensor data fusion approach for target classification. Individual attributes of the unknown target induce local declarations in the form of belief functions by assigning masses to each of the subsets of the frame of discernment. To address possible conflict, a reliability degree, which is essentially the normalized average pairwise discordance index (APDI), is proposed based on the discriminatory power of each evidence source (attribute or feature). The reliability degree is then used as a weighting factor to obtain a weighted average belief function. The weighted average basic probability assignment (BPA) is fused to produce the final BPA. Decisions are taken based on the probability transformation of the final BPA. The flowchart of the proposed method is shown in Figure 4.

The entire flowchart can be summarized using the three building blocks of modeling, reasoning, and decision making. The reasoning block corresponds to the credal level with the decision-making block representing the Pignistic level of the transferable belief model (TBM) [18].

### 3.1. Modeling

#### 3.1.1. Representation of Historical/Training Data

Fuzzy membership functions are used to model every attribute for the various target classes. In other words, the fuzzy membership functions are deployed to estimate the likelihood of the various target classes. The membership functions are built from the statistical information (the mean and the standard deviation) extracted from the training set.

Individual class *i* having an attribute *j* is represented by a triangular membership function using the statistical information. The mean ${\bar{x}}_{ij}$ and the standard deviation $\sigma_{ij}$ for every class $C_{i}\left(i=1,2,\ldots ,M\right)$ and attribute $A_{j}\left(j=1,2,\ldots ,N\right)$ are defined as:

(11) $${\bar{x}}_{ij}=\frac{1}{T}\sum_{t=1}^{T}x_{ijt}$$

(12) $$\sigma_{ij}=\sqrt{\frac{1}{T-1}\sum_{t=1}^{T}(x_{ijt}-{\bar{x}}_{ij}}{)}^{2}$$

**Figure 5:**
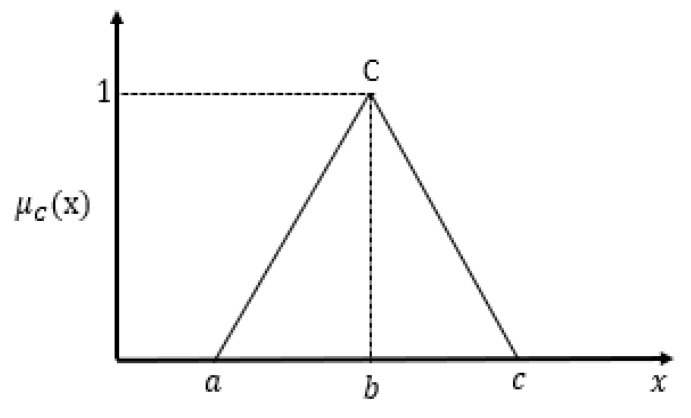
Triangular fuzzy model.

$x_{ijt}$ is the $t^{th}$ sample value given attribute *j* and class *i*. *T* is the sample size for the class. Therefore, for class *i* and a given attribute *j* the triplets for the triangular fuzzy number shown in Figure 5 is defined as

$$a={\bar{x}}_{ij}-\gamma \sigma_{ij}$$

$$b={\bar{x}}_{ij}$$

$$c={\bar{x}}_{ij}+\gamma \sigma_{ij}$$

where γ is a tuning parameter (adjustment factor) as opposed to being set to 2 in [12,13].

#### 3.1.2. Determination of the Reliability Degree

A reliability factor was proposed in [13] and its defined as follows

(13) $$r_{j}=1-\frac{2}{M(M-1)}\sum_{i=1}^{M-1}\sum_{k=i+1}^{M}S_{C}\left(C_{i},C_{k}\right)$$

where, *M* is the number of classes, $S_{C}\left(C_{i},C_{k}\right)$ is the concordance index between class *i* and *k*. We define the reliability degree as the average pairwise discordance index (APDI). Therefore the normalized reliability of source *j* can be expressed as

(14) $$\eta_{j}=\frac{r_{j}}{\sum_{j=1}^{N}r_{j}}$$

The normalization is required to satisfy the constraint imposed by the DS combination rule. Such that

(15) $$\sum_{j=1}^{N}\eta_{j}=1$$

In [13], the reliability factor was not normalized, normalization occur after its combination with the credibility degree before the DS fusion.

### 3.2. Reasoning

The reasoning entails the generation, analysis, and the combination of BPAs. As can be seen, no calculation of credibility is required at the reasoning. Only the (static) reliability obtained at the modeling stage is used. The credibility using the similarity among evidence is with the assumption that a greater proportion of the evidence sources are credible. This assumption may not always be true.

#### 3.2.1. Generation of the Basic Probability Assignment (BPA)

This is where information modeled as belief functions are extracted from sensor measurement. The attribute values of the unknown target are of lower abstraction level, which is mapped into a higher information abstraction level in the form of BPAs. The BPAs are generated based on the similarity between the different attribute values and the fuzzy models(membership functions) obtained from the historical/training data. Due to its simplicity, a similar method used in [11,12,13] is adopted in this work.

#### 3.2.2. Computation of the Weighted Average BPA

Suppose there are *N* evidence sources provided by *N* sensors. For any proposition *A*, a subset of the frame of discernment, the weighted average mass function is a weighted combination of confidence polled from the different evidence sources and it is defined as [20]

(16) $$m_{wae}\left(A\right)=\sum_{j=1}^{N}\eta_{j}*m_{j}\left(A\right)$$

#### 3.2.3. Dempster Shafer (DS) Fusion

Having obtained the weighted average evidence, the next step is to apply the traditional DS Rule of combination on $m_{wae}$ in (N−1) times [19].

### 3.3. Decision Making

This essentially consists of the transformation of the belief function and the application of an appropriate decision rule.

#### 3.3.1. Pignistic Transformation

The final BPA retrieved from the DS fusion cannot be employed directly for decision making, hence, a transformation of the final mass function to probability distribution is required. A well-known probability transformation is the Pignistic probability transformation of the transferable belief model defined as [18]

(17) $$BetP\left(\left\{A\right\}\right)=\sum_{A\subseteq B}\frac{\left|A\cap B\right|}{\left|B\right|}m\left(B\right)$$

B∈F

The ultimate goal of target classification is to assign the unknown target to one of the known classes, thus |A| equals 1, hence, (17) reduces to (18).

(18) $$BetP\left(\left\{A\right\}\right)=\sum_{A\subseteq B}\frac{m(B)}{\left|B\right|}$$

We adopted the Pignistic probability for decision making following the justification of its suitability provided in [30]

#### 3.3.2. Decision Rule

Assign the unknown target to the class with the highest Pignistic probability.

(19) $$A^{*}=arg\;\max_{A\in \Omega }\;BetP\left(\left\{A\right\}\right)$$

#### 3.3.3. Selection of Spread Factor γ

**Figure 6:**
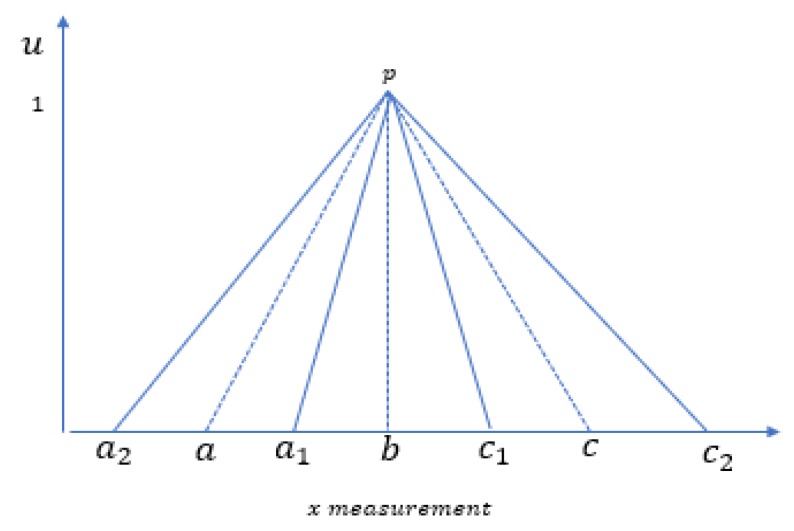
Three models including the upper, the mid, and the lower membership functions.

The following example is used to illustrate the impact of γ on the accuracy of the proposed model on the Iris dataset using only reliability as a weighting factor. Three models are built using 3 different values of γ, as shown in Figure 6 with the triangular fuzzy numbers (TFN1: TFN3). Model1, Model2, and Model3 can be viewed as the lower, the mid, and the upper membership functions, respectively. By applying 5 fold cross-validation 10 times, the associated accuracy with the three different values of γ is shown in Table 1. The focus of this study is not to deploy Type−2 fuzzy set as the likelihood estimation model but demonstrate a type−2 fuzzy set as part of the intuition behind this study.

- Model1: $TFN1=(a_{1},b,c_{1})$, $a_{1}=b-1.5sd$, $c_{1}=b+1.5sd$
- Model2: TFN2=(a,b,c), a=b−2sd, c=b+2sd
- Model3: $TFN3=(a_{2},b,c_{2})$, $a_{2}=b-2.5sd$, $c_{2}=b+2.5sd$

Having discovered that by changing the value of γ, we can alter the decision accuracy of the DS model. The next question is how to select the value of γ. The training set is used to determine a suitable value of γ. With 5-fold cross-validation, we increase γ from 1.5 with a step size of 0.1 to 4.5, and their corresponding accuracies on the training set are recorded. The value of γ that returns the maximum accuracy is selected to build the fuzzy models.

### 3.4. Selection of Evidence Source

In the proposed framework, every attribute measurement is considered as a source of evidence to induce a corresponding belief function. The implication is that the utilization of every attribute obtained from signature and kinematic sets of the targets for characterization will unavoidably lead to high processing costs [31]. The long processing time comes from the combination of the various belief functions using the DS rule of combination. In addition to high processing costs, conflict in evidential reasoning can also be attributed to a large number of evidence sources [23]. Reducing the number of sources is analogous to the challenge of dimensionality reduction in the conventional machine learning algorithm.

Dimensionality reduction is one of the most well-known strategies to remove irrelevant and redundant features. The strategies can be broadly categorized into feature extraction and feature selection [32]. In feature extraction, the original feature space is transformed into a new feature space with a reduced dimension. However, in feature selection, a subset of the original feature space that enhances the performance of the machine learning algorithm is selected. In this study, feature selection is of importance to us for enhanced interpretation. As a result, we will incorporate a preprocessing stage that will involve a reduction of the cardinality of the measurement set based on the significance of each attribute in relation to its discriminatory capability for the various target classes. Only a set of significant attributes is selected as sources of information to produce the basic probability assignment (BPA). In the traditional machine learning, feature selection can be subdivided into two groups [33]:

- *Filter*: Features are ranked based on evaluation criteria independent of learning algorithms. Filter methods have proven to be computationally efficient for feature subset selection.
- *Wrapper*: In wrapper, the ranking of individual features utilizes learning algorithms. Wrapper method is more computationally expensive than the filter methods.

Suppose there are *N* information sources, $I_{j}\left(j=1,\ldots ,N\right)$. The proposed selection method is a filter-based approach that utilizes the average pairwise discordance index(APDI). It is implemented through the following steps:

1. Evaluation of sources using average pairwise discordance index (APDI)
  (20) $$APDI\left(I_{j}\right)=1-\frac{2}{M(M-1)}\sum_{i=1}^{M-1}\sum_{k=i+1}^{M}S_{Conc}\left(Y_{i},Y_{k}\right)$$
  where, *M* is the # classes, and $Y_{i}$ and $Y_{k}$ are class *i* and *k* respectively. $S_{Conc}\left(Y_{i},Y_{k}\right)$ is the concordance index between class *i* and *k*
2. Selection of sources based on certain threshold
  - Compute the mean APDI
  - Select source whose APDI is at least equal to the mean APDI

A pseudo code for the proposed method of selection of information sources is presented in Algorithm 1.
**Algorithm 1** Feature Selection Using APDI**INPUT**: Given a set of information sources $I=[I_{1},\ldots I_{N}]$**OUTPUT**: Selected set of information sources *S*1:S=∅2:**for**j=1≤N**do**3:    Compute the APDI $APDI(I_{j})$4:**End**5:Compute the mean of the APDI $\bar{APDI}$6:**for**j=1≤N**do**7:    **if** $APDI\left(I_{j}\right)\geq \bar{APDI}$ **then**8:        $S=S\cup I_{j}$9:    **else**10:        S=S11:    **endif**12:**endfor**13:**return***S*

## 4. Simulation & Results

### 4.1. Simulation

Four problems consisting of three real datasets and one synthetic dataset were used to demonstrate the capability of the proposed reliability-based multisensor data fusion approach. The performance of the proposed method was compared with the recently proposed method in [13], and Decision Trees (DT) using five-fold cross-validations.

#### 4.1.1. Real Datasets

The three real datasets: IRIS, Wine, and Breast cancer, were obtained from the UCI Machine Learning Repository. Information with respect to the datasets are depicted in Table 2.

#### 4.1.2. Synthetic Dataset

A similar method of generating a synthetic dataset for an airborne target recognition problem for an air surveillance system used in [34] is adopted to illustrate the capability of the proposed method. Each target is described by three features: speed, acceleration, and length. The target belongs to one of the three classes of Commercial plane, Bomber, or Fighter. Recognition is based on a multisensor system to measure the average speed, the maximum acceleration, and the average length. The feature intervals for the various airborne target classes are shown in Table 3. A total of 300 samples were generated with 100 samples for each of the classes based on the information provided in Table 3.

### 4.2. Results

**Figure 7:**
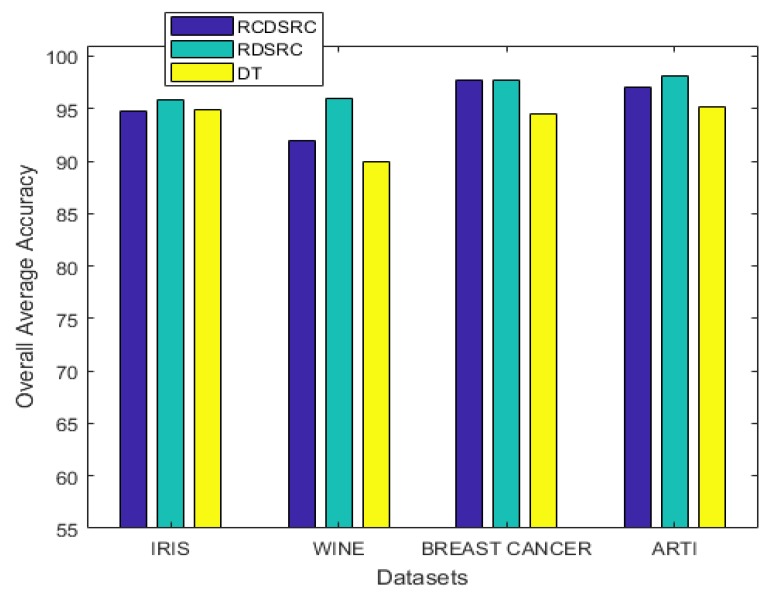
The average accuracy.

We repeated the 5−fold cross validation 10 times, and the average accuracy of each of the different methods is displayed in Table 4 and Figure 7.

**Figure 8:**
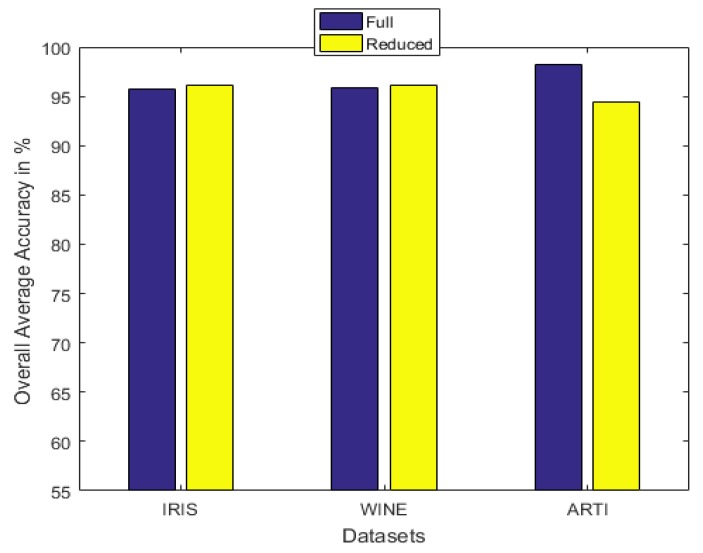
The average accuracy for the full and the reduced datasets.

With the introduction of the proposed attribute selection method, the experimental results are displayed in Table 5, Table 6 and Table 7. The ranking order of the various attributes for the different datasets are shown in Table 5 and their associated APDI displayed in Table 6. The average accuracy for both full and the reduced dataset after repeating the 5 fold cross-validation 10 times is shown in Table 7 and Figure 8.

Acc1 is the accuracy with the full set, while Acc2 is the accuracy with the reduced set. |Fullset| and |Reducedset| are the cardinalities of the full and the reduced sets respectively.

**Figure 9:**
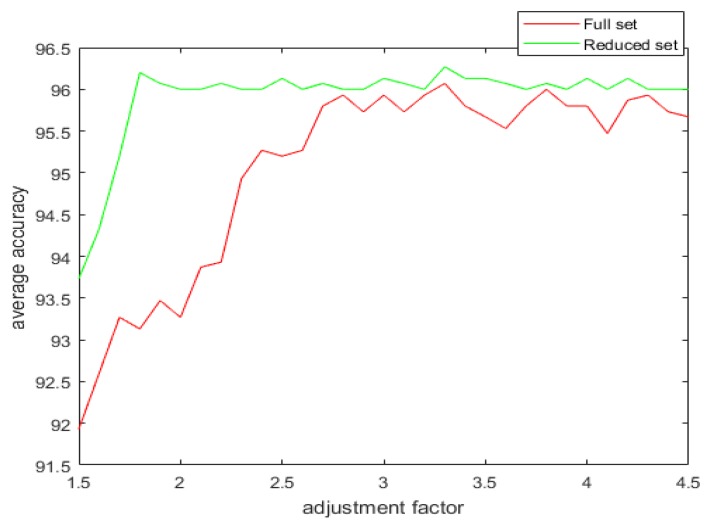
The average accuracy versus the adjustment factor for the full and the reduced Iris dataset.

**Figure 10:**
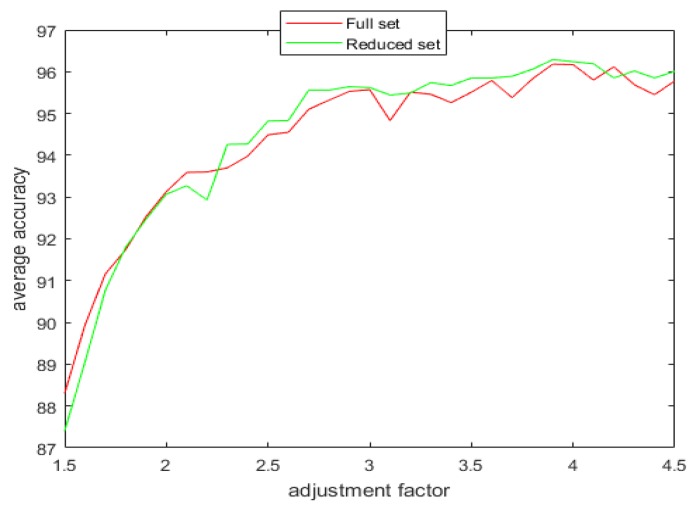
The average accuracy versus the adjustment factor for the full and the reduced Wine dataset.

**Figure 11:**
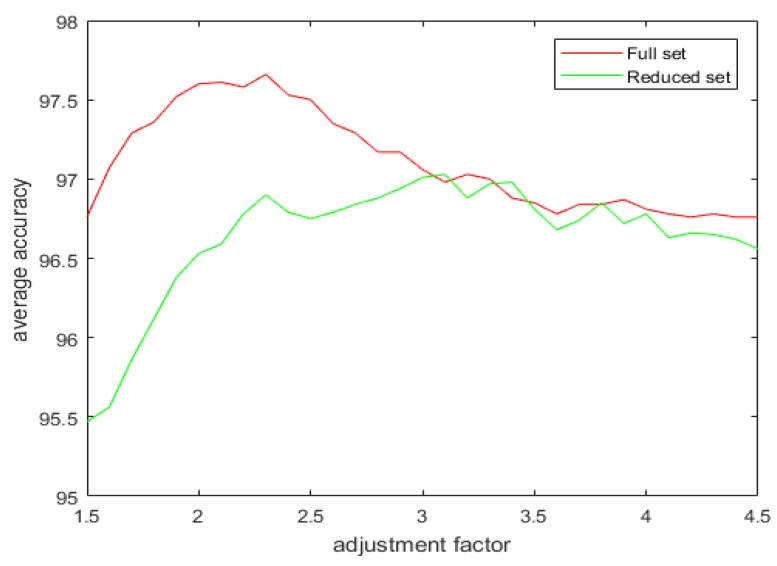
The average accuracy versus the adjustment factor for the full and the reduced Wisconsin Breast Cancer dataset.

**Figure 12:**
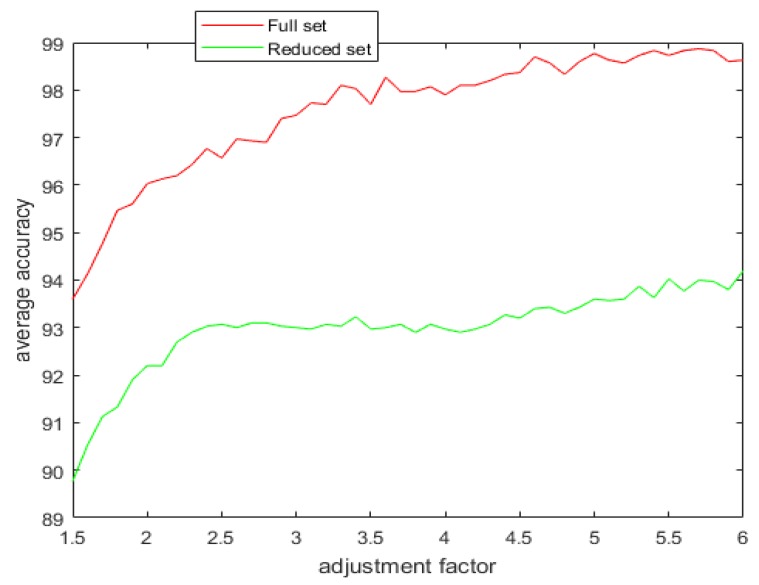
The average accuracy versus the adjustment factor for the full and the reduced artificially generated dataset.

Figure 9, Figure 10, Figure 11 and Figure 12 show the effect of the spread factor on the average recognition accuracy of the proposed method with 5 fold cross validation after 10 trials for both the full and the reduced sets for the different problems.

### 4.3. Discussion

It can be observed from the simulation results that the spread factor is of crucial importance to the decision accuracy of the classification system. It can be seen that the average accuracy of the newly proposed method, RDSRC, is better than the earlier method, RCDSRC, as well as DT. To reduce the number of evidence sources, we propose the APDI as an evaluation index. The average accuracy with the reduced evidence source is better than that of the full evidence source for both Iris and Wine datasets. However, for the Wisconsin breast cancer dataset and the artificially generated data set for the target classification problem, the classification accuracy with the full set is better than that of the reduced set.

## 5. Conclusions

We have proposed a reliability-based multisensor data fusion with application in target classification. This approach fundamentally consists of the representation of the training sets using triangular fuzzy membership functions, the generation of the local declarations in the forms of belief functions by mapping the various attribute measurements into the basic probability assignment (BPA). The various BPAs are preprocessed using the normalized reliability degree based on the goodness/ importance of the attribute to obtain the weighted average BPA. The weighted average BPA is fused with itself using the traditional DS rule of combination to obtain a final declaration (BPA). Then, decisions are made based on the Pignistic probability transformation of the final BPA. It is evident that this approach does not require the computation of the credibility. Through extensive simulations, the average accuracy of the newly proposed method is better than RCDSRC and DT on both the real and artificial datasets. The proposed selection method does not capture redundancy among information sources. The future research effort will be channeled towards incorporating a strategy to handle redundancy among the different sources.

## Figures and Tables

**Table 1:** Average accuracies of the three models.

Model	Model1 γ=1.5	Model2 γ=2.0	Model3 γ=2.5
Accuracy	92.33	93.73	95.67

**Table 2:** Description of datasets.

Datasets	Number of Features	Number of Instances	Number of Classes
Iris	4	150	3
Wine	13	178	3
Breast Cancer	9	699	2
Artificial	3	300	3

**Table 3:** Attribute intervals for the three airborne target classes.

Classes	Average Speed (km/h)	Max Acc (g)	Ave Length (m)
Commercial	[600, 800]	[0, 1]	[25, 65]
Bomber	[400, 700]	[0, 4]	[15, 45]
Fighter	[500, 1000]	[0, 6]	[10, 30]

**Table 4:** Average accuracy.

Datasets	RCDSRC	RDSRC	DT
Iris	94.53	95.80	94.93
Wine	91.91	96.01	89.89
Breast Cancer	97.67	97.69	94.50
Artificial	97.00	98.10	95.10

**Table 5:** Feature ranking using APDI.

Datasets	Ranking Order
Iris	[4 3 1 2]
Wine	[7 12 13 11 10 6 1 4 9 2 8 5]
Breast Cancer	[2 9 3 6 7 4 1 5 8]
Artificial	[3 2 1]

**Table 6:** Corresponding APDI in the same order.

Datasets	APDI Values
Iris	[0.8882 0.8501 0.4355 0.2007]
Wine	[0.6161 0.5562 0.4788 0.4135 0.3961 0.3775 0.3687 0.1663 0.1593 0.1474 0.1351 0.0522 0.0483]
Breast Cancer	[0.6618 0.6568 0.6553 0.4918 0.4347 0.4119 0.4102 0.3749 0.1175]
Artificial	[0.7275 0.6168 0.5053]

**Table 7:** The average accuracy for the full and the reduced datasets.

S/N	Dataset	|*Full Set*|	|*Reduced Set*|	Acc1	Acc2
1	Iris	4	2	95.70	96.07
2	Wine	13	7	95.87	96.13
3	Breast Cancer	9	4	97.70	96.90
4	Artificial	3	2	98.20	93.70
